# Preparing Malaysia for Population Aging through the Advanced Memory and Cognitive Service in Hospital Universiti Sains Malaysia

**DOI:** 10.21315/mjms2023.30.5.1

**Published:** 2023-10-30

**Authors:** Asrenee Ab Razak, Nabilah Abdul Rahman, Mohd Faizal Mohd Zulkifly, Nur Asma Sapiai, Picholas Kian Ann Phoa, Mohamed Faiz Mohamed Mustafar, Sanihah Abdul Halim, Muhammad Ihfaz Ismail, Siti Nurbaya Mohd Nawi, Ahmad Shahril Ab Halim, Hasnetty Zuria Mohamed Hatta, Jafri Malin Abdullah

**Affiliations:** 1 Department of Psychiatry, School of Medical Sciences, Universiti Sains Malaysia, Kelantan, Malaysia; 2 Department of Psychiatry, Hospital Universiti Sains Malaysia, Kelantan, Malaysia; 3 Brain and Behaviour Cluster, Hospital Universiti Sains Malaysia, Universiti Sains Malaysia, Kelantan, Malaysia; 4 Department of Neurosciences, Hospital Universiti Sains Malaysia, Universiti Sains Malaysia, Kelantan, Malaysia; 5 Department of Radiology, Hospital Universiti Sains Malaysia, Kelantan, Malaysia; 6 Department of Internal Medicine, Hospital Universiti Sains Malaysia, Kelantan, Malaysia; 7 Rehabilitation Unit, Hospital Universiti Sains Malaysia, Kelantan, Malaysia

**Keywords:** Geriatrics, aging, cognition, memory, neurocognitive disorder, dementia

## Abstract

Improving healthcare and living conditions has led to an increase in life expectancies and challenges of population aging in Malaysia. The Advanced Memory and Cognitive Service builds on integrated healthcare among multidisciplinary specialists to provide holistic and patient-centred healthcare. The service treats older adults experiencing neurocognitive impairment as well as young individuals with complex neurocognitive disorders and thoroughly screens asymptomatic individuals at high risk of developing neurocognitive disorders. This early intervention strategy is a preventive effort in the hope of reducing disease burden and improving quality of life to prepare Malaysia for the forthcoming population aging.

## Introduction

Malaysia is facing the inevitable challenge of population aging, characterised by a growing proportion of older individuals. The declining death rates, longer life expectancies, and improvements in healthcare and living conditions contribute to this demographic shift. The Department of Statistics Malaysia also observed that Malaysia’s aging population is growing rapidly. In the first quartile of 2023, Malaysia’s population observed a net increase of 1.6%, wherein the number of live births increased by 8.9% and the number of recorded deaths reduced by 11.7% (1). This finding corresponds with the speculation that more than 15% of Malaysia’s population will be above 65 years old by 2050 (2).

An aging population can face several social, economic and healthcare challenges. One consequence of an aging population is the rising prevalence of major neurocognitive disorders (MNCDs), previously known as dementia. MNCDs are defined as significant decline in at least one of the cognitive domains (i.e. executive function, complex attention, language, learning, memory, perceptual-motor or social cognition) that is severe enough to interfere with independence and daily life (3). A recent epidemiological study by Ganapathy et al. (4) reported that 8.5% of Malaysian individuals aged 60 years old and above suffered from MNCDs. Although their prevalence is lower than that of diseases such as diabetes, hypertension, hyperlipidaemia and heart disease, the Malaysia Aging and Retirement Survey indicated that MNCDs restricted older people’s daily activities more than the latter (5). In fact, dementia was the second and third leading cause of disability burden among women and men aged 80 years old and above, respectively (6).

As the absolute and relative number of older people increases, it is logical to expect an increase in the number of older patients admitted to hospitals. Consequently, new services related to their care must be developed and implemented (7). Insights into and attention to the treatment of neurocognitive disorders are essential for improving specialised care. Individualised care and targeted interventions can lower the risk of MNCDs (8). A multidisciplinary approach is critical for addressing the complex nature of MNCDs, which involve neurological and psychological factors. Thus, we launched the Advanced Memory and Cognitive Service in May 2023 at the Hospital Universiti Sains Malaysia (HUSM). This service employs a holistic clinical model in which all medical professionals (i.e. psychiatrists, neurologists, geriatricians, clinical psychologists, radiologists and occupational therapists) and active participation from caregivers provide an integrated and interdisciplinary assessment of patients with or at risk of neurocognitive disorders.

### Current Practice in HUSM

The Memory Clinic at HUSM was established in 2007 to meet the psychological needs of older patients. The unit operates under the Department of Psychiatry, where services are provided primarily by psychogeriatric psychiatrists, assisted by trainee psychiatrists. We received outpatient referrals from all HUSM departments and other external centres for patients with cognitive impairment for thorough assessment, consultation, treatment, and management. The Memory Clinic also conducted community psychogeriatric services via home visits based on patients’ needs and outreach programs such as in-community support groups and dementia screening programmes. Although this effort has enhanced the management of neurocognitive disorders, patient experiences and healthcare quality may be compromised because of a lack of integration with other health specialties and services. Thus, incorporating various health services such as neurological assessments, advanced radiological techniques and occupational therapies is essential in improving the quality of healthcare services.

### The Advanced Memory and Cognitive Service

The Advanced Memory and Cognitive Service is a new, integrated, one-stop centre for the specialised care of patients with complex neurocognitive disorders. Besides geriatrics, the Advanced Memory and Cognitive Service now also accepts referrals for patients aged < 65 years old with neurocognitive impairment to screen for young-onset dementia and individuals who are cognitively unimpaired but potentially at risk (e.g. family history of MNCDs and subjective cognitive and memory decline). Effective screening and early consultation with specialists can expedite the management of MNCDs in young individuals, thereby increasing their potential for better health outcomes (9). This is achieved by incorporating advanced imaging techniques and clinical assessments to identify specific disorders and their progression.

Clinical psychologists play a central role in conducting neuropsychological assessments and cognitive enhancement interventions. Initially, cognitive screening is performed, including administration of the Mini-Mental State Examination or the Montreal Cognitive Assessment. The outcome of this screening determines the standardised testing required. Later, other neuropsychological assessments (i.e. tests of general cognitive functioning, memory, language, visual-perceptual ability, attention, executive functions and processing speed) are administered to aid in diagnosis, cognitive profiling, identifying strengths and weaknesses, and creating a treatment plan for rehabilitation.

Beyond conventional pencil-and-paper neuropsychological testing, imaging techniques, such as electroencephalography (EEG), are helpful tools for distinguishing patients with normal and abnormal brain activity. Clinicians should consider the amplitude, frequency and power of EEG rhythms as predictive markers (10, 11). Additionally, precise identification of different types of dementia through conventional and advanced sequence magnetic resonance imaging (MRI) allows for the timely initiation of targeted molecular therapies. Follow-up imaging is necessary to evaluate disease progression and treatment responses.

In the Advanced Memory and Cognitive Service, standard MRI brain protocol for dementia cases include axial T1-weighted (T1W), T2-weighted, 3D fluid-attenuated inversion recovery, axial susceptibility-weighted imaging, diffusion-weighted imaging, oblique-coronal 3D T1W turbo field echo of the whole brain with angle follow long axis temporal lobe, T2 coronal high resolution 3 mm angled through temporal lobe, 3D Arterial Spin Labeling (ASL), and magnetic resonance angiogram 3D Time-of-Flight Circle of Willis. If suspicious lesions are identified, patients undergo contrast studies or diffusion tensor imaging (DTI) to provide additional insight into the underlying neuropathology.

ASL is a non-invasive perfusion neuroimaging technique primarily used in MRI to measure cerebral blood flow in the brain. ASL provides quantitative information on brain perfusion and the amount of blood delivered to a given volume of brain tissue per unit of time (12). This is crucial for understanding brain function, which can be affected by neurological disorders. In the study of dementia, ASL can help researchers and clinicians assess cerebral perfusion patterns. Changes in blood flow can indicate early stage dementia. It also aids in differentiating between various types of dementia (e.g. Alzheimer’s disease, vascular dementia and frontotemporal dementia) for better planning of treatment strategies and monitoring patient responses to therapy (12).

DTI is another specialised neuroimaging technique used at our centre. In dementia, white matter tracts, which are responsible for transmitting signals between different brain regions, are often damaged (13). DTI can assess the integrity of these tracts by measuring the direction and speed of water diffusion within them and differentiate between various types of dementia. For example, it can distinguish between Alzheimer’s disease, which primarily affects the gray matter and vascular dementia, which often involves white matter damage due to vascular issues.

Following the investigations, cognitive rehabilitation is planned for patients and their family members, including restorative and compensatory approaches. Computerised cognitive rehabilitation systems, such as CogniPlus, provide individualised cognitive training. Training is tailored to an individual’s level of impairment and the difficulty level increases upon assessment. Patients must attend multiple sessions of cognitive rehabilitation until they achieve their performance goals. The Brain Stimulation Clinic is a new service offered to our patients. Clinically, repetitive transcranial magnetic stimulation (rTMS) is used as an alternative treatment approach for patients with brain disorders. This non-invasive technique has the potential to reduce symptoms of depression in dementia and improve cognitive performance in older adults with brain disorders (14, 15). Eligible patients must participate in several rTMS sessions and neuropsychological measurements monitor their progress before and after treatment. After the completion of rTMS treatment, follow-ups are performed and rTMS maintenance sessions are scheduled to prevent relapse.

## Conclusion and Future Perspective

Although the trend towards older populations is primarily irreversible, government choices and community activities can influence its course and effects. The effects of population aging extend beyond older adults and affect many facets of the economy and society. Thus, the healthcare sector should take timely critical measures to enable communities to adapt to and benefit from population aging to prevent the tremendous costs imposed on future social, economic, fiscal and health-related expenditures. Integrated and multidisciplinary healthcare services for patients with neurocognitive impairment can accelerate the management of MNCDs and improve their prognoses and quality of life.

## Figures and Tables

**Figure 1:**
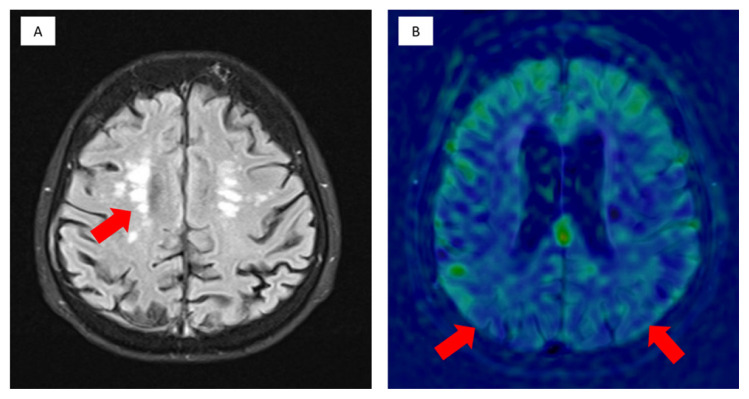
A 74-year-old lady with gradual memory impairment MRI (A) axial Fluid-attenuated inversion recovery (FLAIR) shows multiple foci of deep white matter hyperintensities, and (B) fused ASL perfusion with FLAIR axial imaging reveals significantly reduced cerebral blood flow of the bilateral parietal

**Figure 2:**
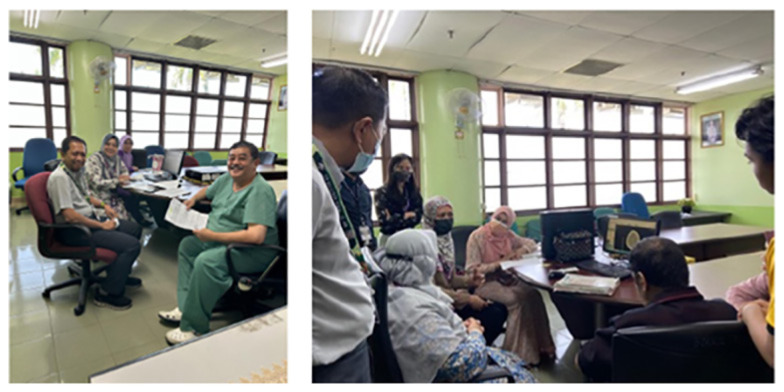
Photographs taken at the Advanced Memory and Cognitive Service on 23 May 2023, attended by patients and experts from psychiatry, neurology, clinical psychology, geriatric medicine, radiology and occupational therapy

## References

[1] Department of Statistics Malaysia (DOSM) (2023). Demographic statistics Malaysia first quarter 2023.

[2] Department of Statistics Malaysia (DOSM) (2020). Current population estimates, Malaysia, 2020.

[3] Sachdev PS, Blacker D, Blazer DG, Ganguli M, Jeste DV, Paulsen JS (2014). Classifying neurocognitive disorders: the DSM-5 approach. Nat Rev Neurol.

[4] Ganapathy SS, Sooryanarayana R, Ahmad NA, Jamaluddin R, Abd Razak MA, Tan MP (2020). Prevalence of dementia and quality of life of caregivers of people living with dementia in Malaysia. Geriatr Gerontol Int.

[5] Asian Development Bank (ADB) (2023). Malaysia Ageing And Retirement Survey Wave 2 (2021–2022) survey report.

[6] Institute for Public Health (IPH) (2017). Malaysian Burden of Disease and Injury Study 2009–2014.

[7] Mafauzy M (2000). The problems and challenges of the ageing population of Malaysia. Malays J Med Sci.

[8] Rossor MN, Fox NC, Mummery CJ, Schott JM, Warren JD (2010). The diagnosis of young-onset dementia. Lancet Neurol.

[9] Panegyres P, Berry R, Burchell J (2016). Early dementia screening. Diagnostics.

[10] Babiloni C, Arakaki X, Bonanni L, Bujan A, Carrillo MC, Del Percio C (2021). EEG measures for clinical research in major vascular cognitive impairment: recommendations by an expert panel. Neurobiol Aging.

[11] Babiloni C, Arakaki X, Azami H, Bennys K, Blinowska K, Bonanni L (2021). Measures of resting state EEG rhythms for clinical trials in Alzheimer’s disease: recommendations of an expert panel. Alzheimer’s and Dementia.

[12] Rahmat K, Haris Phuah A, Ramli N, Muhammad Gowdh NF, Chan WY, Tan MP (2022). Neuroimaging in dementia syndromes. Neurol Asia.

[13] Staffaroni A, Elahi F, McDermott D, Marton K, Karageorgiou E, Sacco S (2017). Neuroimaging in dementia. Semin Neurol.

[14] Burley CV, Burns K, Lam BCP, Brodaty H (2022). Nonpharmacological approaches reduce symptoms of depression in dementia: a systematic review and meta-analysis. Ageing Res Rev.

[15] Bonotis K, Anargyros K, Liaskopoulos N, Barlogianni A-M (2022). Evaluation of memory performance in patients with brain disorders following rTMS treatment: a systematic review. Clin Neurophysiol.

